# Depressive Symptomatology among Norwegian Adolescent Boys and Girls: The Patient Health Questionnaire-9 (PHQ-9) Psychometric Properties and Correlates

**DOI:** 10.3389/fpsyg.2017.00887

**Published:** 2017-06-08

**Authors:** Jasmina Burdzovic Andreas, Geir S. Brunborg

**Affiliations:** Department of Substance Use, Norwegian Institute of Public HealthOslo, Norway

**Keywords:** depression, adolescents, PHQ-9, Norway, cross-cultural comparison

## Abstract

This study explored the potential contribution of the Diagnostic and Statistical Manual for Mental Disorders (DSM-IV)-based Patient Health Questionnaire-9 item (PHQ-9) instrument to the developmental epidemiology research in Norway, by examining depressive symptoms in a school sample of adolescents (*N* = 846). The average PHQ-9 scores were 6.89 (SD = 5.13) for girls, and 4.57 (SD = 3.98) for boys; 8.5% of girls and 2.6% of boys were classified into the originally proposed categories indicative of Major Depressive Disorder (MDD; PHQ-9 scores ≥ 15). Multi-group confirmatory factor analysis (CFA) confirmed a single-factor structure for the PHQ-9 with solid psychometric properties and high internal consistency for both genders. However, even though configural equality was observed, there was no evidence for metric or scalar equality across genders, warranting further investigation of measurement equivalence for the current Norwegian version of the PHQ-9. We observed no major associations between the PHQ-9 scores and adolescent religion or immigrant background. Further, school grade, not living together with both biological parents, and diagnosed chronic illness were differently associated with elevated depressive symptoms for boys and girls. Finally, high residential instability, perceived low SES, school dissatisfaction, lack of close friendships, history of suicide attempts and self-harm, and elevated emotional problems were all significantly and consistently associated with greater depression for both genders. Overall, the PHQ-9 appears to be a promising research tool, potentially offering clinically-relevant classification of adolescent self-reported depressive symptomatology in addition to the symptom severity captured by continuous scores. Nevertheless, further investigation concerning the observed measurement non-equivalence, as well as the comprehensive validation and comparison against the gold standard is required before the PHQ-9 is to be used for diagnostic screening in Norway.

## Introduction

Depression in children and adolescents is far from infrequent or inconsequential (Birmaher et al., 1996; Lewinsohn et al., 1998; Merikangas et al., 2010; Rohde et al., 2012; Thapar et al., 2012). Even though many children recover, early-onset depression remains a potent risk factor for subsequent mental health problems and other negative outcomes (Harrington et al., 1990; Weissman et al., 1999; Kovacs et al., 2016). In addition, early gender differentiation has also been observed, where adolescent girls tend to have both elevated symptoms and a different developmental course of depression (Hankin et al., 1998; Twenge and Nolen-Hoeksema, 2002; Dunn and Goodyear, 2006; Dekker et al., 2007; Essau et al., 2010). Accurately detecting early depression symptoms, and detecting them accurately for boys and girls is thus a public health imperative. In that regard, brief self-reports may be especially useful, as such screeners can rapidly identify “at-risk” youth in need of further evaluation and possibly treatment.

This may especially be true for Norway, where the 2014 Public Health report states that “mental disorders are a major health problem for children and adolescents in Norway today” (Norwegian Institute of Public Health, 2014). Despite these clearly identified issues and the general focus on adolescent health and development, the use of diagnostically-informative measures in research practice has been somewhat limited in Norway. For example, multiple Norwegian studies investigating early depression (Sund et al., 2003, 2011; Lundervold et al., 2013; Larsson et al., 2016) have utilized various versions of the Mood and Feelings Questionnaire (MFQ; Angold et al., 1995). Its wide use in studies of developmental epidemiology notwithstanding, the MFQ is fairly extensive (34 items for the full and 13 items for the short version), rendering it not necessarily the best *brief* instrument. And even though the MFQ can theoretically aid in clinical screening, its cut-offs are not fully established or necessarily even recommended^1^. Other reports have used shorter and thus more practical instruments; for example the 12-item and 5-item Symptom Checklists (SCL-12, and SCL-5) (Heyerdahl et al., 2004; Derdikman-Eiron et al., 2012; Myklestad et al., 2012), and a 6-item Depressed Mood Inventory (Wichstrøm, 1999; Abebe et al., 2016), all of which appear to measure anxiety and depression and to have been derived from the 25-item Hopkins Symptom Checklist (HSCL) for adults (Derogatis et al., 1974). However, it is not entirely clear how appropriate these derivations may be for the assessment of adolescents, or how well they differentiate between anxiety and depression given that they tend to conflate the two into “anxiety-depression” (Heyerdahl et al., 2004) and are meant to measure general “psychological” or “global mental distress” (Tambs and Moum, 1993; Strand et al., 2003; Myklestad et al., 2012). In addition, the items, responses, cut-off scores, and clinical interpretation of these HSCL derivations appear to vary from study to study in Norway (i.e., from version to version; Heyerdahl et al., 2004; Derdikman-Eiron et al., 2012; Abebe et al., 2016). Finally, psychiatric symptoms among Norwegian adolescents have also been evaluated using the Strengths and Difficulties Questionnaire (SDQ) and its various subscales (Rønning et al., 2004; Indredavik et al., 2005; Goodman et al., 2011). Despite its many advantages and widespread international use, the 25-item SDQ is also somewhat long, and it ideally requires the children's, parental, and teachers' reports for a complete evaluation. Thus, any potential large-scale screening based on multiple SDQ reports/informants may be both impractical and costly. The use of SDQ for such purposes may additionally be questionable, given the related cultural considerations (Heiervang et al., 2007, 2008), as well as its somewhat limited ability to detect mental health disorders in general (Brøndbo et al., 2011), and depressive symptomatology in particular. For example, it is not clear how well the SDQ Emotional Problems subscale differentiates between anxiety and depression, as both classes of problems appear to be assessed under the general heading of “emotional” disorders (Goodman et al., 2000a,b).

Thus, the public health initiatives and the related research and clinical practice in Norway could benefit from a self-report instrument designed to swiftly and effectively screen specifically for early-onset depression based on the common and internationally-validated criteria. The 9-item Patient Health Questionnaire (PHQ-9) adolescent version may be especially well-suited for such purposes (Kroenke et al., 2001; Johnson et al., 2002). First, the PHQ-9 has only 9 items, and it requires only the youth self-reports. The instrument was originally developed to assess symptoms of depression in accordance to the *Diagnostic and Statistical Manual of Mental Disorders, 4th* edition (DSM-IV) criteria, but it also corresponds to the more recent DSM-5 criteria (American Psychiatric Association, 2016). Further, the PHQ-9 has been shown to be a valid tool in detecting depression among adolescents across various cultures and settings (Adewuya et al., 2006; Richardson et al., 2010; Fatiregun and Kumapayi, 2014; Tsai et al., 2014). Most importantly, the PHQ-9 also captures depression severity, as it provides both continuous scores and clinically-meaningful classification of depressive symptomatology. Availability of continuous scores ensures no loss of variability, and thus may be both recommended and preferred in research, whereas categorical classification may be more meaningful in clinical practice and assessment. Ideally, a self-report instrument should serve both purposes, and the PHQ-9 for adolescents theoretically does so at the international level. For example, the PHQ-9 was successfully used to estimate the prevalence of Major Depressive Disorder (MDD) in Chinese and Nigerian school samples (Fatiregun and Kumapayi, 2014; Tsai et al., 2014), in addition to the US community and primary care samples (Richardson et al., 2010; Rhew et al., 2016). Such apparent cultural robustness strengthens the potential advantages of this instrument in multiple large scale national and cross-cultural comparisons.

In conclusion, there is a need for a brief and internationally validated instrument that captures both the severity and corresponding clinical categories of self-reported depressive symptomatology in Norwegian adolescent populations. Such an instrument would strengthen both the research and clinical practice in Norway, while simultaneously opening the door to cross-cultural and cross-national monitoring, evaluation, and comparison. Perhaps the PHQ-9—with its brief format, simple self-report administration, and attractive scoring features—can fill this need. This report offers the first step in that direction, by examining the PHQ-9 basic properties and correlates in a sample of Norwegian adolescents. Specifically, we examined: (1) the basic psychometric properties of the PHQ-9, including measurement equivalence (invariance) by gender, and (2) severity and correlates of depressive symptoms among Norwegian middle- and high-school students as measured by the PHQ-9.

## Methods

### Sample and procedures

The sample comprised middle- and high-school students enrolled in a mixed-methods short-term longitudinal study primarily focusing on substance use among Norwegian youth. Seven schools in the vicinity of the Norwegian capital were approached for study participation, with the goal of complete enrollment in grades 8 through 12. A total of 1,326 students from the five assenting schools were approached for survey participation. Middle-school students' participation was predicated upon their own assent and parental consent, whereas high-school students (i.e., those older than 16) consented themselves. A modest contribution was made to each participating classroom (approximately 100–120 Euros), while the teachers who helped out with data collection were reimbursed with a modest honorarium. Of the consented 943 (71.1%) students, 884 (93.7%) participated in the baseline assessment conducted in the Fall of 2014, where they completed a computer-administered questionnaire during their regular class time under teacher supervision.

Students were relatively evenly distributed across middle school (17.9% in grade 8, 15.0% in grade 9, and 18.3% in grade 10) and high-school grades (23.9% in grade 11 and 24.9% in grade 12). Approximately half of the participants were boys (46.3%), and the majority had no immigrant background (80.7% reported both parents born in Norway, and 91.8% were Norwegian-born themselves). This report utilized data from the baseline assessment. Outliers (*n* = 9) and cases with incomplete responses on the depression scale items (*n* = 29) were excluded, resulting in an analytical sample of 846 students.

The study was approved by the Data Protection Official for Research/Norwegian Centre for Research Data (NSD, case #39513). Additional descriptions of the sample and study procedures are provided elsewhere (Brunborg et al., 2017).

### Measures

The student questionnaire assessed a range of developmentally-relevant characteristics from all levels of human ecology (Bronfenbrenner, 1979). All instruments were based on the internationally validated and commonly used measures, which were translated and modified for the Norwegian context as needed.

#### Depressive symptomatology

Students reported their symptoms of depression during the last 7 days on the 9-item Patient Health Questionnaire (PHQ-9) adapted for use with adolescents (Kroenke et al., 2001; Johnson et al., 2002) and as recommended for research and clinical evaluation by the American Psychiatric Association (2016). The PHQ-9 uses the DSM-IV diagnostic criteria to assess depressive symptomatology (i.e., sleep, concentration, and energy problems, low self-esteem, anhedonia, etc.) on a 4-point scale ranging from 0 (“not at all”) to 3 (“nearly every day”), (Kroenke et al., 2001; Kroenke and Spitzer, 2002).

In addition to its utility as a short screener, the PHQ-9 also captures depression severity. Overall scale scores are computed as a sum of the 9 items (possible range 0–27), and the prorated scores can be obtained as long as there are at least 7 items with valid responses (American Psychiatric Association, 2016). The corresponding severity categories were originally defined as *none* (PHQ-9 scores 0–4), *mild* (PRQ-9 scores 5–9), *moderate* (PHQ-9 scores 10–14), *moderately severe* (PHQ-9 scores 15–19), and *severe* (PHQ-9 scores 20–27; Richardson et al., 2010). Adolescents with the PHQ-9 scores of 15 or above (i.e., those classified as exhibiting *moderately severe*, or *severe* depressive symptomatology) may be of particular clinical concern, as they are likely to meet the diagnostic criteria for Major Depressive Disorder (MDD) with 95% specificity (Kroenke et al., 2001; Tsai et al., 2014).

#### Demographics

Students reported their *gender, school grade* (8 through 12), *religion*, and whether they and their parents were born in Norway. Participants were classified as *native-born* if they were born in Norway, and without *immigrant background* if both of their parents were also Norwegian-born. In addition, students reported their residence circumstances, including *residential instability* (i.e., the number of school changes due to the family move) and whether they currently live with their *intact biological family*. Finally, the *perceived low social status* was measured by the MacArthur Scale of Subjective Status—Youth version (Goodman et al., 2001), where the participants placed their family along the Norwegian socio-economic ladder ranging from those families who “have it best” (coded “1”) to those who are “the worst off” (coded “10”).

#### Psycho-social characteristics

Students completed the 5-item School Connectedness Scale (McNeely et al., 2002). The original items (e.g., “I feel like I am part of this school”) utilized Likert-type response options ranging from 1 (“completely agree”) to 5 (“completely disagree”). The items were averaged to compute the scale score (Cronbach's α = 0.83, possible range 1–5) such that greater scores reflected the risk factor of greater *school disconnectedness*. Students also reported if they feel they have at least one *close friend*. Additional health problems were assessed with two items asking about *lifetime suicide attempts* and *self-harm*, and with a single item asking about the presence of a *diagnosed chronic illnesses*. Finally, participants also completed the 5-item Emotional Problems subscale from the Strengths and Difficulties Questionnaire (SDQ; Goodman and Goodman, 2009) previously used in Norwegian samples (Heiervang et al., 2008; Goodman et al., 2011; Bøe et al., 2016). The original SDQ 3-point responses were summed up to compute the Emotional Problems scores (Cronbach's α = 0.74, possible range 0–10). In addition, participants were classified into those with clinical-level emotional problems (i.e., scoring at or above the cut-off score of 6) vs. rest, using the SDQ norms for Norwegian adolescents (Rønning et al., 2004; Van Roy et al., 2006).

#### Statistical analyses

The initial set of analyses focused on the basic psychometric properties and structure of the PHQ-9, which we examined for the entire sample, and separately for boys and girls. Next, before an instrument is used to compare levels of a latent variable (e.g., depression) between groups, it is important that the instrument is established as measurement equivalent for such groups (also referred to as measurement *invariant*). To that extent, we used multi-group confirmatory factor analysis (CFA) to examine the PHQ-9 measurement equivalence for boys and girls as described by Byrne (2012).

Specifically, we examined: (a) configural equality to test whether the factor structure is equal for boys and girls by fitting a model where factor loadings and intercepts were allowed to vary between the two groups; (b) metric equality, to test whether items are interpreted in the same way for both boys and girls by restricting factor loadings to be equal, but letting intercepts vary between the two groups; and (c) scalar equality, to test whether the response scale is used in the same way by boys and girls by restricting factor loadings to be equal and restricting all but one intercept to be equal for the two groups, and full scalar equality by restricting all factor loadings and all intercepts to be equal for the two groups. Direct comparisons (e.g., tests of differences in means) between two groups are valid only if scalar equality holds. The robust maximum likelihood estimator was used because we did not assume multivariate normality for the items. The Satorra-Bentler Chi-square test (S-B χ^2^) was used to test statistically whether the CFA models were different. In addition, the root-mean square error of approximation (RMSEA), the comparative fit index (CFI), the standardized root mean square residual (SRMR), and Akaike information criterion (AIC) were used to assess model fit. Suggested cut-off points indicating adequate fit for the RMSEA, CFI and SRMR are ≤0.08, >0.90, and ≤0.05, respectively (Byrne, 2012). The AIC has no cut-off points, but lower AIC suggests better fit.

Finally, we examined divergent/convergent validity and the associations between the adolescent PHQ-9 measures and other psycho-social characteristics. These analyses were based on simple, unadjusted regression models. CFA was performed using Mplus, and all other analyses were performed in STATA statistical software.

## Results

### Sample characteristics

The results shown in Table 1 indicate that even though ours was a convenience sample, it appeared highly representative of the Norwegian adolescent population. This is not surprising, given the relative homogeneity of Norwegian society. For example, the basic socio-demographic characteristics, such as residing with both biological parents (approximately 2/3 in our sample vs. “62 per cent for 17 year-olds” for Norway as a whole) and having immigrant background (i.e., 18.6% with at least one parent born outside of Norway from our sample vs. 16.3% “born in Norway of two foreign-born parents and four foreign-born grandparents” for Norway as a whole) appear reflective of the official Norwegian population estimates (Statistics Norway, 2016, 2017). However, even though approximately 2.4% of the Norwegian population self-identifies as Muslim, this proportion was 4.4% in our sample. Whether this was a realistic departure from the Muslim representation among Norwegian adolescents specifically is not known. Most importantly, in terms of psychological adjustment, our sample appears congruent with other youth community-samples from Norway. For example, the lifetime prevalence of suicidal attempts (defined as those who reported such attempts plus those who “refused to answer”) was 8.8% in our sample, vs. 8.2% observed in a representative sample of high-school students (Wichstrøm, 2000). Finally, the average SDQ Emotional Problems scores from our sample, as well as the proportion of clinical-level cases were comparable to the estimates obtained from several representative samples in Norway (Rønning et al., 2004; Van Roy et al., 2006). For example, Van Roy et al. (2006) classified a total of 12.2% of younger adolescents and 13.4% of older adolescents into the SDQ Emotional Problems clinical range in 2006, as compared to 16.4% of our sample in early-to-middle adolescence in 2014.

**Table 1:** Sample characteristics; by gender.

**Variables**	**Boys (*n* = 387)**	**Girls (*n* = 459)**
	***M*** **(SD) or %**	***M*** **(SD) or %**
**DEMOGRAPHICS**		
**School grade (8 through 12)**	10.2 (1.4)	10.2 (1.4)
**Religion**		
None declared	38.0	22.6
Christianity	53.0	70.2
Islam	4.1	4.6
Other	4.9	2.6
**Residential instability**		
1 move/school change	14.7	13.1
2 or more moves/school	13.7	12.2
changes		
**Intact biological family (Yes)**	66.7	66.4
**Born in Norway (Yes)**	90.4	90.0
**Immigrant background (Yes)**	19.1	18.3
**Perceived low SES**	3.7 (1.8)	4.1 (1.8)
**PSYCHO-SOCIAL CHARACTERISTICS**		
**School dissatisfaction**	1.6 (0.61)	1.7 (0.71)
**Close friend (Yes)**	93.3	90.4
**Ever attempted suicide**		
Yes	2.7	4.2
Don't know/Will not respond	4.9	5.6
**Ever self-harmed**		
Yes	8.4	19.4
Don't know/Will not respond	3.3	5.6
**Chronic illness diagnosis (Yes)**	21.6	21.5
**SDQ Emotional problems (continuous scores)**	2.0 (1.8)	3.8 (2.4)
**SDQ Emotional problems (clinical positive)**	5.94	25.3

*Shown are means (standard deviations) for continuous variables, and proportions (%) for categorical variables*.

### Item statistics and distributional properties

**Figure 1:**
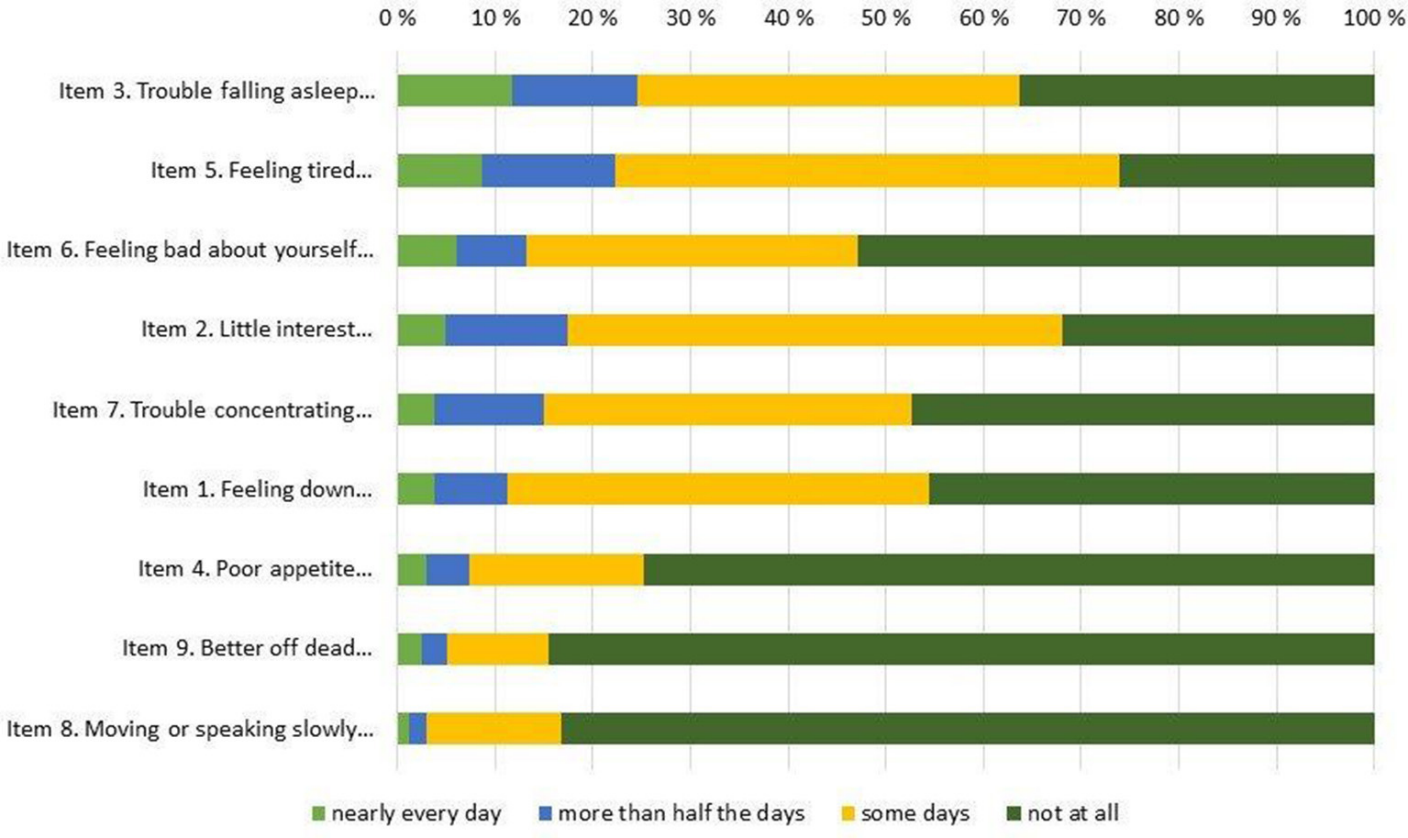
PHQ-9 item response frequency distribution total sample.

**Figure 2:**
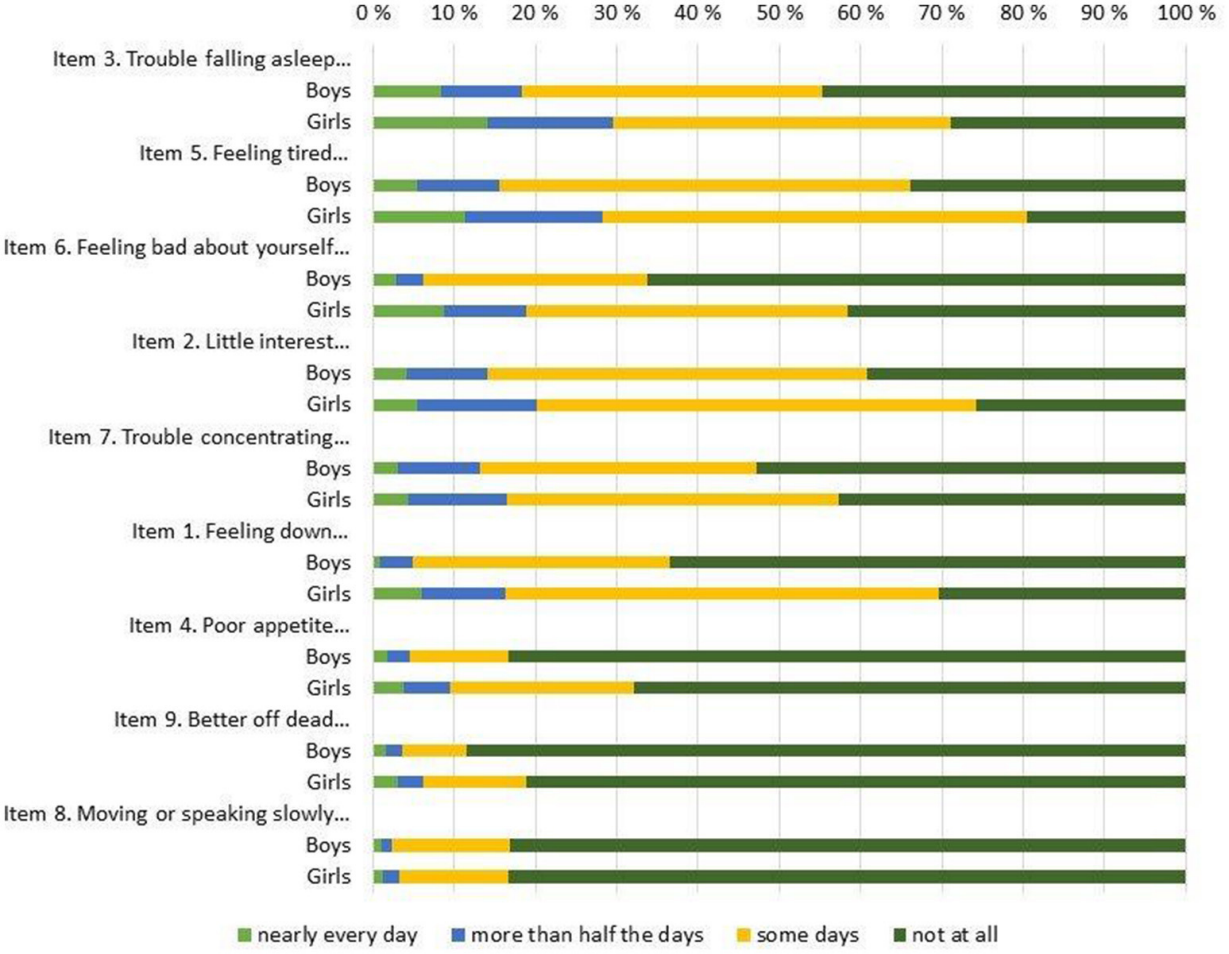
PHQ-9 item response frequency distribution by gender.

The overall response rate was very high for all items, with 6 or fewer omissions on all items, save for item #8 (i.e., “Moving or speaking so slowly that other people could have noticed…”) where 14 participants failed to respond. Table 2 shows basic descriptive statistics for all 9 individual items (top of Table 2) and for the entire scale (bottom of Table 2), both for the entire sample and for boys and girls separately. In addition, the response distributions for individual items are shown in Figure 1 (for the entire sample) and Figure 2 (by gender), ordered by the prevalence of the most severe response category (i.e., “nearly every day”). The Figure 1 pattern indicates that the sleep problems (item #3), energy loss (item #5), low self-esteem (#6), and anhedonia (item #2) were the items endorsed with greatest frequency, whereas movement problems (item #8) and suicidal ideation (item #9) were the items endorsed with lowest frequency. Figure 2 shows discrepancies in the item response patterns between boys and girls. For example, nearly 60% of girls endorsed the low self-esteem item (#6) in some form, as opposed to only 34% of boys. In general, girls appeared more likely to endorse all items, save for item #8, as evident in the average scores (shown in Table 2) and response distribution (shown in Figure 2).

**Table 2:** The PHQ-9 item and scale descriptives.

	**Patient Health Questionnaire 9 items (PHQ-9) Adolescent version**		**Item M (SD)**		
			**Total (*N* = 846)**	**Boys (*n* = 387)**	**Girls (*n* = 459)**
1.	Feeling down, depressed, irritable, or hopeless		0.69 (0.76)	0.42 (0.61)	0.92 (0.80)
2.	Little interest or pleasure in doing things		0.90 (0.79)	0.79 (0.78)	1.00 (0.79)
3.	Trouble falling asleep, staying asleep, or sleeping too much		1.00 (0.98)	0.82 (0.93)	1.15 (0.99)
4.	Poor appetite, weight loss, or overeating		0.35 (0.70)	0.23 (0.59)	0.43 (0.77)
5.	Feeling tired, or having little energy		1.05 (0.86)	0.87 (0.80)	1.20 (0.88)
6.	Feeling bad about yourself, or that you're a failure or that you've let yourself or your family down		0.66 (0.85)	0.43 (0.69)	0.86 (0.92)
7.	Trouble concentrating on things like school work, reading, or watching TV		0.71 (0.81)	0.63 (0.79)	0.78 (0.82)
8.	Moving or speaking so slowly that other people could have noticed		0.21 (0.52)	0.20 (0.50)	0.21 (0.54)
9.	Thought that you would be better off dead, or of hurting yourself in some way		0.23 (0.61)	0.17 (0.52)	0.28 (0.67)
			**Scale Descriptives**		
	**PHQ-9 overall scores**	M (SD)	5.83 (4.78)	4.57 (3.98)	6.89 (5.13)
		Range	0–27	0–24	0–27
		Skewness (s.e.)	1.48 (0.08)	1.55 (0.12)	1.35 (0.11)
		Kurtosis (s.e.)	2.86 (0.17)	3.69 (0.25)	2.16 (0.23)

Response options: 0, “not at all”; 1, “some days”; 2, “more than half the days”; 3, “nearly every day.”

Distributions for the originally proposed PHQ-9 diagnostic categories are shown in Table 3. As would be expected, the overall PHQ-9 scores did not follow the normal distribution, as the majority of our participants reported no or only mild depressive symptomatology (also see Figure 1, 2 for individual item distribution). The average PHQ-9 score was 6.89 (5.13) for girls and 4.57 (3.98) for boys, while 8.5% of girls and 2.6% of boys were classified into the original PHQ-9 categories indicative of MDD (i.e., PHQ-9 scores ≥15).

**Table 3:** Originally proposed PHQ-9 severity categories; total sample and by gender.

**PHQ-9 Depression severity categories**	**Total (*N* = 846) %**	**Boys (*n* = 387) %**	**Girls (*n* = 459) %**
None (PHQ-9: 0–4)	46.8	57.9	37.8
Mild (PHQ-9: 5–9)	36.1	32.8	38.8
Moderate (PHQ-9: 10–14)	11.3	6.7	15.3
Moderately severe (PHQ-9: 15–19)	3.4	1.6	5.0
Severe (PHQ-9: 20–27)	2.4	1.0	3.5
PHQ-9 clinical-level (PHQ-9 ≥ 15) Moderately severe/severe depression	5.8	2.6	8.5

### Confirmatory factor analysis and measurement equivalence

The results from the confirmatory factor analysis (CFA)—where all PHQ items were set to load on one latent factor (i.e., “depression”) according to its theoretical conceptualization—are shown in Table 4, including standardized factor loadings and fit indices. The CFA confirmed a single-factor solution, with standardized factor loadings ranging from 0.51 (item #8, “Moving or speaking so slowly that other people could have noticed”) to 0.77 (item #6, “Feeling bad about yourself, or that you're a failure or that you've let yourself or your family down”) for the entire sample. This single-factor solution displayed acceptable fit to the data (bottom of Table 4). Cronbach's alpha for the items was 0.86. Conceptually identical CFA results were obtained for boys and girls when analyzed separately (Table 4), including the single factor solutions, and the poorest and best performance exhibited by the #8 “movement” item (factor loading_Boys_ = 0.40, factor loading_Girls_ = 0.60) and #6 “self-esteem” item (factor loading_Boys_ = 0.71, factor loading_Girls_ = 0.77). The model fit the data adequately for both girls and boys (bottom of Table 4); Cronbach's alpha_Boys_ = 0.81; Cronbach's alpha_Girls_ = 0.88.

**Table 4:** Confirmatory factor analysis for a single-factor solution for the PHQ-9 items; total sample and by gender.

**PHQ-9 items**		**Standardized factor loadings**		
		**Total (*N* = 846)**	**Boys (*n* = 387)**	**Girls (*n* = 459)**
1.	Feeling down, depressed, irritable, or hopeless	0.73	0.63	0.75
2.	Little interest or pleasure in doing things	0.56	0.46	0.61
3.	Trouble falling asleep, staying asleep, or sleeping too much	0.62	0.56	0.62
4.	Poor appetite, weight loss, or overeating	0.63	0.63	0.61
5.	Feeling tired, or having little energy	0.67	0.55	0.70
6.	Feeling bad about yourself, or that you're a failure, or that you've let yourself or your family down	0.77	0.71	0.77
7.	Trouble concentrating on things like school work, reading, or watching TV	0.61	0.57	0.64
8.	Moving or speaking so slowly that other people could have noticed	0.51	0.40	0.60
9.	Thought that you would be better off dead, or of hurting yourself in some way	0.62	0.64	0.63
**FIT INDICES**				
	S-B χ^2^ (*df*)	148.8 (27)	74.6 (27)	98.6 (27)
	RMSEA	0.073	0.067	0.076
	CFI	0.93	0.91	0.94
	SRMR	0.04	0.049	0.04

The results from multi-group equality testing are presented in Table 5. Metric equality was assessed by comparing the configural model (Model A) with a model where factor loadings were restricted to be equal (Model B). The S-B χ^2^ test was not statistically significant. The RMSEA and CFI values did not change substantially and remained within the acceptable range. The AIC was higher for Model B compared to Model A, and the SRMR was higher and outside of the acceptable range for Model B, indicating worse absolute fit compared to Model A. Overall, the results do not support the assumption of metric equality across genders. This also means that scalar equality was not supported. In practice, such a set of results implies that further formal tests of gender differences in our sample should not be conducted without caution. This multi-group equality testing was repeated with the weaker item #8 and/or the highly skewed item #9 omitted from the CFA. The results showed again that metric and scalar equivalence was not supported in either case.

**Table 5:** Tests of PHQ-9 measurement equivalence across gender.

**Model**	**Comparison model**	**S-B χ^2^ (*df*)**	**S-B Δ χ^2^ (*df*)**	***P***	**RMSEA**	**CFI**	**SRMR**	**AIC**
A. Basic model (configural equality)	–	171.6 (54)			0.072	0.926	0.045	14714
B. Full metric equality	A.	187.3 (63)	8.75 (9)	0.44	0.068	0.922	0.093	14733
C. Partial scalar equality	B.	238.5 (70)	65.33 (7)	<0.001	0.075	0.894	0.100	14783
D. Full scalar equality	B.	247.8 (71)	77.95 (8)	<0.001	0.077	0.889	0.100	14793

*Shown are results from the CFA analyses examining PHQ-9 equivalence between boys and girls. As we did not assume multivariate normality, the robust maximum likelihood estimator (MLR) was used. Model fit was assessed by the Satorra-Bentler (S-B) adjusted χ^2^ difference test, root mean square error of approximation (RMSEA), the comparative fit index (CFI), standardized root mean square residual (SRMR), and Akaike's information criterion (AIC)*.

### Adolescent psycho-social characteristics and depressive symptomatology

Table 6 documents the associations between the adolescents' psycho-social characteristics and the PHQ-9 continuous scores, which were investigated separately for boys and girls because of the aforementioned results concerning measurement non-equivalence. These associations indicate that the basic demographic characteristics—including religious affiliation and parental or adolescent immigrant background—appeared to have minimal associations with depressive symptomatology among adolescents from our sample. Demographic characteristics associated with greater depressive symptomatology were the school grade (for girls only), high residential instability and the low perceived SES (for both boys and girls). A set of more specific risk-factors and health characteristics—such as school dissatisfaction, lack of close friendships, history of suicide attempts and self-harms, and elevated emotional problems as measured by the SDQ subscale—were consistently and significantly associated with depressive symptomatology across both genders (Table 6). Specifically, for both boys and girls, the explicit report of lifetime suicide attempt was associated with a roughly 10-point increase in the PHQ-9 scores (Table 6). Similarly, each 1-point increase in the SDQ Emotional Problems scores was associated with the significant increases of 1.17-point and 1.33-point in the PHQ-9 scores, or standardized regression coefficients of *r* = 0.54 and *r* = 0.63, *p* < 0.001 for boys and girls, respectively. An identical pattern of results was obtained when the SDQ clinical categories were examined: membership in the SDQ Emotional Problems category was associated with an approximately 6-point increase in the PHQ-9 scores for both genders. Among these psycho-social characteristics, the only one exhibiting gender differential was the self-reported diagnosis of chronic illness (Table 6), such that poorer physical health was significantly associated with greater symptoms of depression for boys but not for girls from our sample.

**Table 6:** Adolescent depressive symptomatology as a function of psycho-social characteristics; crude associations by gender.

**Variables**	**Boys (*n* = 387)**	**Girls (*n* = 459)**
	***b* (s.e.)**	***b* (s.e.)**
**DEMOGRAPHICS**		
**School grade (8 through 12)**	0.18 (0.14)	0.50 (0.16)^***^
**Religion**		
None declared^a^	–	–
Christianity	−0.75 (0.43)	−0.74 (0.58)
Islam	−0.13 (1.04)	−0.21 (1.23)
Other	−0.58 (0.97)	−2.33 (1.56)
**Residential Instability**		
0 moves/school changes^a^	–	–
1 move/school change	0.97 (0.56)	1.07 (0.71)
2 or more moves/school	2.27 (0.58)^***^	2.57 (0.73)^***^
changes		
**Intact biological family**		
No ^a^	–	–
Yes	−1.18 (0.42)^**^	−0.24 (0.51)
**Born in Norway**		
No^a^	–	–
Yes	−0.41 (0.69)	−0.12 (0.79)
**Immigrant background**		
No^a^	–	–
Yes	0.19 (0.51)	−0.21 (0.62)
**Perceived low SES**	0.27 (0.11)^*^	0.32 (0.13)^*^
**PSYCHO-SOCIAL CHARACTERISTICS**		
**School dissatisfaction**	2.47 (0.30)^***^	3.48 (0.29)^***^
**Close friend**		
Other^a^	–	–
At least one close friend	−3.44 (0.79)^***^	−3.81 (0.79)^***^
**Ever attempted suicide**		
No^a^	–	–
Yes	9.16 (1.18)^***^	10.24 (1.07)^***^
Don't know/Will not respond	2.03 (0.89)^*^	5.00 (0.94)^***^
**Ever self-harmed**		
No^a^	–	–
Yes	5.20 (0.69)^***^	5.61 (0.54)^***^
Don't know/Will not respond	3.22 (1.08)^**^	5.77 (0.93)^***^
**Chronic illness diagnosis**		
No^a^	–	–
Yes	1.49 (0.49)^**^	0.98 (0.57)
**Emotional problems (SDQ scores)**	1.17 (0.09)^***^	1.33 (0.07)^***^
**Emotional problems (SDQ clinical)**		
No^a^	–	–
Yes	6.09 (0.79)^***^	6.22 (0.47)^***^

*Shown are the unstandardized regression coefficients (i.e., b) from crude regression models examining the associations between each individual characteristic and adolescent depressive symptomatology as measured by the PHQ-9 continuous scores. For categorical predictors, the reference group is noted by superscript ^a^*.

* *p < 0.05*.

** *p < 0.01*.

*** *p < 0.001*.

## Discussion

Our results appear consistent with previous international reports examining depression in adolescent school samples using the PHQ-9 instrument, including the school samples of Chinese and Nigerian adolescents (prevalence of *moderately severe/severe depression* = 5.2 and 5.1%, respectively; Fatiregun and Kumapayi, 2014; Tsai et al., 2014) and the community samples of American adolescents (*moderately severe/severe depression* prevalence = 6.5%, even though this study used a somewhat different clinical classification algorithm; Rhew et al., 2016). Most importantly, our estimate of approximately 6% prevalence of clinically-elevated symptoms is relatively congruent with other recent reports of current depressive symptomatology among Norwegian adolescents, ranging from 2.6% for MDD and 6.3% for depressive disorder not otherwise specified during the 2-month window (Sund et al., 2011), to the 11% prevalence of the less specific “high depressive symptoms” during the past week (Abebe et al., 2016). Nevertheless, these estimates should be interpreted as preliminary given that we used the cut-off scores established internationally but not in Norway, and the sample which was not necessarily representative of all Norwegian adolescents.

Our CFA results confirmed that the PHQ-9 measures adolescent depression as a unidimensional theoretical construct. Relatively high factor loadings (i.e., all loadings ≥0.40) and solid fit indices were observed for the entire sample, and for boys and girls separately. One item (e.g., “Moving or speaking so slowly that other people could have noticed”) consistently showed poorer, yet still acceptable performance. It is possible that the wording and/or meaning were difficult for younger participants to comprehend, as evident in the relatively high number of missing responses on this particular item. Specifically, this question asked about what other people could have noticed, which may be confusing for youngest participants. Refinement or alternative wording of this item may improve the scale performance.

In agreement with previous evidence that girls tend to exhibit greater depressive symptomatology than boys starting around the age of 13 (Hankin et al., 1998; Wichstrøm, 1999; Twenge and Nolen-Hoeksema, 2002), and with other Norwegian reports demonstrating substantive gender differences in measurements of adolescent depression (Lundervold et al., 2013), girls from our sample appeared to have greater depression problems. However, we did not proceed to perform formal tests of gender differences because we could not fully establish metric or scalar equality for the PHQ-9 instrument across genders. This means that girls and boys cannot be directly compared in terms of the PHQ-9 scores without reservations, contrasting previous international reports of the PHQ-9 measurement invariance by gender and age (Yu et al., 2012; Petersen et al., 2015). However, our results may not be out of place in a Norwegian context, where only partial measurement-equivalence for gender was reported for the SDQ instrument in adolescent populations (Bøe et al., 2016). Clearly, the causes and implications of these non-equivalence results should be investigated further when it comes to the use of PHQ-9 (and possibly other instruments for which measurement equivalence was not fully tested before use) in Norway. Nevertheless, it should also be noted that these findings do not preclude utilization of the PHQ-9 in research practice, as long as the analyses are stratified by gender (Bøe et al., 2016).

Finally, we examined divergent and convergent validity, as well as the associations between the PHQ-9 scores for boys and girls separately. Given that the PHQ-9 was not previously used in Norway, we only had general expectations that the direction and magnitude of these associations would be comparable to those from other Norwegian studies on adolescent depression. This was generally the case. We found no strong evidence for the association between depression in adolescents and their basic demographic characteristics such as religion or immigrant status. Such a pattern may be reflecting the somewhat inconclusive state of knowledge regarding the mental health among adolescent immigrants in Norway (Abebe et al., 2014). Other putative risk factors—including age, not living together with both biological parents, and diagnosed chronic illness—were associated with depressive symptomatology differently for boys and girls. Older age appeared to be a risk factor for depression among girls only, while not living with both biological parents or chronic illness appeared to be risk factors only among boys. In contrast, high number of moves and school changes, perceptions of one's family as poor by Norwegian standards, social isolation and lack of close friendships, history of suicide attempts and self-harms, and elevated SDQ emotional problems were uniformly and significantly associated with elevated depression for both genders. Similar patterns—for example, the associations between elevated depression and residential instability, low SES, not living with both biological parents, school dissatisfaction and lack of close friendships (Sund et al., 2003; Myklestad et al., 2012)—were observed in other Norwegian adolescents samples. More importantly, our results demonstrated the high convergence between the PHQ-measures with other theoretically related constructs such as suicidality or impairments in emotional adjustment (Goodman and Goodman, 2009; Hawton et al., 2012; Silverstone et al., 2015), where the standardized regression coefficients with the SDQ Emotional problems scores exceeded 0.5 for both genders. They also suggest the potential effectiveness of the PHQ-9 in preliminary identification of youth at high risk for depression using the large-scale epidemiological surveys.

The current study is limited by several factors, including its convenience sample and reliance on self-reports for all indicators. We also used a somewhat shorter time reference and assessed the PHQ-9 depressive symptomatology during the last 7 days as recommended by the American Psychiatric Association (2016). Most importantly, our adolescent PHQ-9 self-reports were not validated against the external diagnostic criteria, such as the official psychiatric diagnoses for example. However, it should be noted that the PHQ-9 has been internationally validated against various diagnostic interviews in multiple adolescent studies (Richardson et al., 2010; Allgaier et al., 2012; Ganguly et al., 2013; Tsai et al., 2014), with some reports even using the PHQ-9 itself as a gold standard against which to validate other measures of depression in community youth samples (Rhew et al., 2016). Nevertheless, external validation of the PHQ-9 against diagnostic interviews would have strengthened our study, especially because we used somewhat conservative criteria and because there may be cultural, ethnic, and national variations in depressive symptomatology and corresponding clinical cut-offs (Kroenke and Spitzer, 2002; Richardson et al., 2010; Allgaier et al., 2012; Ganguly et al., 2013; Jaber et al., 2015). Full validation, including the validation of the originally suggested cut-offs and associated diagnostic categories, is therefore necessary before any clinical evaluation is to be undertaken using the PHQ-9 among Norwegian youth.

Despite these limitations, the PHQ-9 appears to be a promising research tool, potentially offering clinically-relevant classification of adolescent depressive symptomatology in addition to the symptom severity captured by continuous scores. Most importantly, given the internationally validated and streamlined clinical criteria, the use of PHQ-9 has the potential to advance not only national, but also cross-national assessments and comparisons of depressive symptomatology among youth populations. As such, we encourage further exploration of the PHQ-9 adolescent instrument in developmental epidemiology research and in studies of general adolescent health and development. Nevertheless, further study of the hereby observed measurement non-equivalence, as well as a comprehensive validation against the proper diagnostic criteria are required before the PHQ-9 is to be used for youth psychiatric screening in Norway.

## Ethics statement

This study was carried out in accordance with the recommendations of the Norwegian Centre for Research Data (NSD; http://www.nsd.uib.no/nsd/english/pvo.html; Protocol#39513) with informed consent from all participants or guardians.

## Author contributions

The present report was drawn from a larger adolescent development project, directed by JBA and GSB. Both JBA and GSB contributed to study design, development of research questions, data analyses, and writing. Both authors approved the final manuscript.

### Conflict of interest statement

The authors declare that the research was conducted in the absence of any commercial or financial relationships that could be construed as a potential conflict of interest.

## Footnotes

^1^Angold, A. MFQ Message from the Author. Available online at http://devepi.duhs.duke.edu/.%5Cinstruments%5CMFQ%20user.pdf
